# A novel anti-mycobacterial function of mitogen-activated protein kinase phosphatase-1

**DOI:** 10.1186/1471-2172-10-64

**Published:** 2009-12-17

**Authors:** Benny KW Cheung, Howard CH Yim, Norris CM Lee, Allan SY Lau

**Affiliations:** 1 Cytokine Biology Group, Department of Paediatrics and Adolescent Medicine, LKS Faculty of Medicine, The University of Hong Kong, Pokfulam, Hong Kong Special Administrative Region, PR China

## Abstract

**Background:**

*Mycobacterium tuberculosis *(MTB) is a major cause of morbidity and mortality in the world. To combat against this pathogen, immune cells release cytokines including tumor necrosis factor-α (TNF-α), which is pivotal in the development of protective granulomas. Our previous results showed that Bacillus Calmette Guerin (BCG), a mycobacterium used as a model to investigate the immune response against MTB, stimulates the induction of TNF-α via mitogen-activated protein kinase (MAPK) in human blood monocytes. Since MAPK phosphatase-1 (MKP-1) is known to regulate MAPK activities, we examined whether MKP-1 plays a role in BCG-induced MAPK activation and cytokine expression.

**Results:**

Primary human blood monocytes were treated with BCG and assayed for MKP-1 expression. Our results demonstrated that following exposure to BCG, there was an increase in the expression of MKP-1. Additionally, the induction of MKP-1 was regulated by p38 MAPK and extracellular signal-regulated kinase 1 and 2 (ERK1/2). Surprisingly, when MKP-1 expression was blocked by its specific siRNA, there was a significant decrease in the levels of phospho-MAPK (p38 MAPK and ERK1/2) and TNF-α inducible by BCG.

**Conclusions:**

Since TNF-α is pivotal in granuloma formation, the results indicated an unexpected positive function of MKP-1 against mycobacterial infection as opposed to its usual phosphatase activity.

## Background

Tuberculosis (TB) remains a major cause of morbidity and mortality in the world, especially in the developing countries [1]. The disease is caused by *Mycobacterium tuberculosis *(MTB) and approximately one third of the world's population has been infected by this pathogen. In a recent report, World Health Organization (WHO) estimated that there are 9.2 million new TB cases around the world in 2006 [1].

In response to MTB infection, induction of cytokines by immune cells is an important defense mechanism. The infected macrophages secrete intercellular signaling factors, proinflammatory cytokines, to mediate the inflammatory response leading to the formation of granuloma and induction of T-cell mediated immunity [2]. In order to understand TB pathogenesis, signaling pathways induced by mycobacteria have long been a subject of interest. Mitogen activated protein kinases (MAPKs) including extracellular signal-regulated kinase 1 and 2 (ERK1/2), p38 MAPK, and c-Jun N-terminal kinase (JNK) have been implicated as important cellular signaling molecules activated by mycobacteria [3]. Previous reports have shown that p38 MAPK and ERK1/2 are required in the induction of TNF-α expression in human monocytes infected with *M. tuberculosis *H37Rv [4]. We have further revealed the significant role of MAPKs in the signal transduction events of mycobacterial activation of primary human blood monocytes (PBMo) leading to cytokine expressions via the interaction with PKR [5]. However, the subsequent events as to how MAPK is regulated and how such regulation affects cytokine production in response to mycobacteria remain to be elucidated.

Since MAPKs are activated by phosphorylation, dephosphorylation of MAPKs seems to be an efficient process to inactivate their activities. It can be achieved by specific protein kinase phosphatases which can remove the phosphate group from MAPKs. Examples of these phosphatases include tyrosine phosphatases, serine/threonine phosphatases, and dual-specificity phosphatases (DUSPs). Some DUSPs are also known as MAPK phosphatases (MKPs) [6-8]. Currently, there are at least 10 MKPs identified, while MKP-1 is the most studied member of the family. The regulatory role of MKP-1 on cytokine induction is best demonstrated by MKP-1 knock-out (KO) macrophages in response to lipopolysaccharide (LPS), a cell wall component of Gram-negative bacteria. MKP-1 KO macrophages showed prolonged phosphorylation of p38 MAPK and JNK as well as increased production of TNF-α in response to LPS treatment [9]. Consistent with these results, another group further revealed that LPS-treated MKP-1 KO bone marrow-derived macrophages show increased AP-1 DNA-binding activity [10]. Also, they showed that LPS-induced MKP-1 expression is dependent on myeloid differentiation factor 88 (MyD88) and TIR domain-containing adaptor inducing IFN-β (TRIF) [10], thus demonstrating the role of MKP-1 in signal transduction.

Not only LPS, other TLR inducers including CpG, peptidoglycan, poly IC, and Pam_3_Cys can regulate cytokine expressions including TNF-α, IL-10 via MKP-1 activities [10,11]. In these processes, MKP-1 serves to mitigate the undesirable effects of septic shock and maintain organ functions by restraining the inflammatory responses following bacterial infection. Another example of MKP-1 function is the immune response to *Staphylococcus aureus *(*S. aureus*), a Gram positive bacteria. There are higher levels of cytokine production including TNF-α, IL-6, and MIP-1α in MKP-1 KO mice infected with *S. aureus *[12]. Also, the mice would have a rapid development of multi-organ dysfunction as well as faster mortality rate upon challenge with heat-killed *S. aureus *[12]. Taken together, these results suggest that MKP-1 protects the host from overactivation of the immune system in response to Gram negative or Gram positive bacteria.

In the past, it was believed that different MKP/DUSP family members have overlapping functions. However, the emergence of DUSP2 turned the concept up side down [13]. It was shown that DUSP2 behaves differently and is opposite to the function as stated above. In DUSP2 KO cells, they produced less inflammatory mediators, implying that DUSP2 may play a role in mediating instead of limiting inflammation. For instances, when DUSP2 KO macrophages were treated with LPS, there were less TNF, IL-6, nitric oxide, IL-12-producing cells when compared to those of the wild type counterparts [13]. When the DUSP2 KO bone marrow-derived mast cells were first sensitized with immunoglobulin E (IgE) receptor (FcεRI) and then stimulated with dinitrophenol-heat stable antigen, they produced lower TNF mRNA levels, diminished IL-6 production, less phosphorylation of ERK1/2, p38 MAPK, and less transcriptional activities by Elk1 and NFAT-AP-1 [13].

These unexpected positive regulations of immune cell functions by DUSP2 have been hypothesized to be due to crosstalks between MAPKs [13]. Stimulation of KO mast cells and macrophages showed increases in phosphorylation of JNK. Moreover, inhibition of JNK by small molecule inhibitors showed increases in phosphorylation of ERK [13]. The authors also showed that there were physical interactions of DUSP2 with ERK2, DUSP2 with JNK2, as well as DUSP2 and p38 MAPK after stimulation of the cells with dinitrophenol-heat stable antigen. Nevertheless, the details of the crosstalks between MAPKs and phosphatases need further investigation. Thus, the MKP family plays a critical role in the regulation of immune responses.

Innate immune response protects the host from MTB infection by secretion of cytokines including TNF-α in immune cells. Meanwhile, MAPK is one of the critical proteins in the regulation of immunity and cytokine expression. Since MAPK is regulated by MKP-1 in response to LPS and the activation of MAPK is important in BCG-induced cytokine expression, we ***hypothesize ***that MKP-1 plays a critical role in the immune regulation of BCG in human monocytes. We examined the involvement of MKP-1 in BCG-induced MAPK activation and its consequent cytokine expression. Here, we present evidences that MKP-1 plays an unexpected role in the regulation of cytokine induction by BCG through its control of MAPK phosphorylation.

## Results

### Different inducers show differential induction of MKP-1

**Figure 1:**
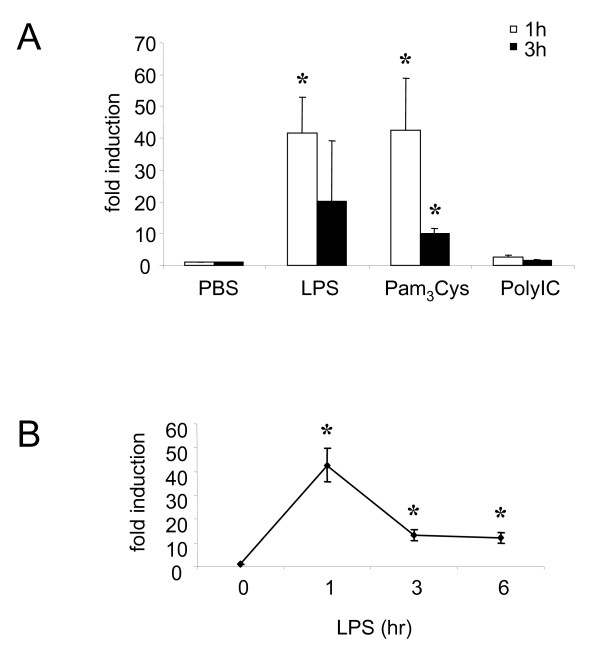
**Different inducers show differential induction of MKP-1**. (A) Primary human blood monocytes were stimulated with LPS (50 ng/ml), Pam3Cys (50 ng/ml), or Poly IC (100 μg/ml) for 1 (open bars) and 3 (black bars) hours. Phosphate buffered saline (PBS) was used as a reagent control. RNA was harvested and MKP-1 mRNA was measured by QPCR method. (B) Primary human monocytes were treated with LPS (50 ng/ml) for the indicated time points and RNA samples were harvested. MKP-1 levels were studied by using QPCR. Independent experiments were done on blood cells from 4 different donors, and the results are shown as mean ± SD. * = *p *< 0.05.

It has been reported that many inducers including growth factors, LPS, peptidoglycan, and dexamethasone can stimulate the expression of MKP-1 in human macrophages, microglia, mast cells or fibroblasts [6]. To investigate the role of different TLR inducers in MKP-1 induction process in human blood monocytes, the level of MKP-1 mRNA was measured by quantitative polymerase chain reaction (QPCR) method. PBMo were isolated from primary human blood mononuclear cells and stimulated with Pam_3_Cys (TLR2 agonist), poly IC (TLR3 agonist), or LPS (TLR4 agonist) for 1 and 3 hours. Following exposure to Pam_3_Cys or LPS, there were significant inductions of MKP-1 mRNA levels within 1 hour of treatment (Figure 1A). These effects on MKP-1 induction continued for 3 hours post-treatment with Pam_3_Cys (Figure 1A). In contrast, poly IC did not induce MKP-1 (Figure 1A). The results indicate that different inducers showed differential up-regulation of MKP-1 expression.

### LPS induces MKP-1 transcription

LPS has been extensively used to demonstrate the role of MKP-1 in immune response both *in vivo *and *in vitro *[9,12]. To establish a foundation for interpretation of subsequent experimental results, LPS was used as a positive control for the induction of MKP-1 expression. To determine the levels of MKP-1 in response to LPS, kinetics of MKP-1 transcription were determined by QPCR. There was a significant induction of MKP-1 mRNA, which peaked as early as 1 hour upon LPS stimulation, and the levels gradually decreased over a course of 6 hours. These results showed that LPS induced MKP-1 expression (Figure 1B).

### BCG induces MKP-1 expression

**Figure 2:**
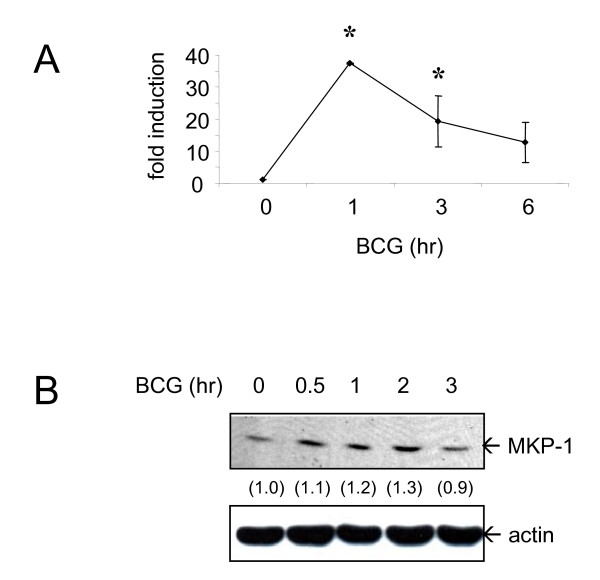
**BCG induces MKP-1 expression**. (A) Human monocytes were treated with BCG (MOI = 1 CFU/cell) for the indicated time points and RNA was harvested. MKP-1 level was assayed by using QPCR. Results are shown as mean ± SD from 3 different donors. * = *p *< 0.05. (B) Cells were treated as in (A) for the indicated time points and proteins were collected. MKP-1 protein levels were measured by Western blotting. Independent experiments were done on monocytes from 3 different donors and one representative set of results is shown. The intensities of the protein bands were determined by using Bio-Rad Quantity One imaging software. The intensities of MKP-1 were normalized to the corresponding actin. The values in parenthesis are the relative normalized intensities compared to those of untreated cells.

Next, to demonstrate the induction of specific phosphatases by BCG, kinetics of MKP-1 expression in PBMo was studied by using QPCR during BCG treatment. Similar to the results produced by LPS, upon the addition of BCG (MOI = 1 CFU/cell), there was a significant induction of MKP-1 mRNA within 1 hour of BCG treatment as determined by Taqman probe specific for MKP-1 (Figure 2A). The effects lasted for at least 6 hours (Figure 2A).

To examine whether the changes of protein production were in parallel to that of the mRNA levels, the protein levels of MKP-1 were measured by Western blotting. In response to BCG, PBMo produced the MKP-1 protein as early as 30 minutes after treatment. The protein levels were maintained for 2 hours and dropped to basal levels at 3 hours (Figure 2B). The results demonstrated that there was MKP-1 induction in response to BCG activation in human monocytes.

### BCG-induced MKP-1 expression is dependent on p38 MAPK and ERK1/2

**Figure 3:**
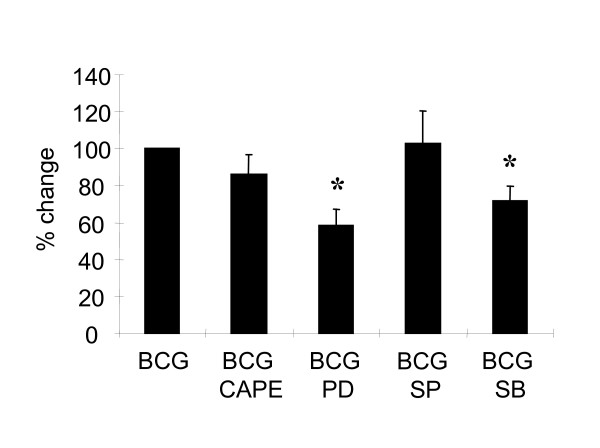
**BCG-induced MKP-1 expression is dependent on p38 MAPK and ERK1/2**. Human monocytes were pretreated with several inhibitors including PD98059 (20 μM), SB203580 (100 nM), SP600125 (100 nM), and CAPE (5 μg/ml) for 1 hour. BCG (MOI = 1 CFU/cell) was then added for 1 hour and RNA was harvested. Levels of MKP-1 were measured by QPCR. Percentage change was defined as the percentage of the fold induction of (BCG + inhibitor) over the fold induction of BCG without the inhibitor. Sample of BCG was set as 100% for comparisons. Independent experiments were performed on blood cells from 4 different donors and the results are shown as mean ± SD. * = *p *< 0.05.

It has been shown that inhibition of p38 MAPK either by specific inhibitor or siRNA reduced the expression of MKP-1 in LPS- or peptidoglycan-treated macrophages [14]. To determine the mechanisms involved in the BCG-induced MKP-1 expression, PBMo were pretreated with several inhibitors including PD98059 (inhibitor for MAP kinase kinase [MEK] or ERK1/2), SB203580 (inhibitor for p38 MAPK), SP600125 (inhibitor for JNK), and CAPE (inhibitor for NF-κB) for 1 hour. A range of concentrations of each inhibitor was used to test their optimal concentrations and effects on cell viability and kinase inhibitions. BCG was added afterwards and total RNA was harvested. The results demonstrated that, with the inhibition of ERK1/2 and p38 MAPK activities by their corresponding relatively specific inhibitors, MKP-1 expressions were significantly reduced (Figure 3). In addition, using higher dose of SB203580, we showed that the inhibition is increased further (data not shown). On the contrary, pretreatment of the cells with CAPE and SP600125 did not affect the induction of MKP-1 by BCG (Figure 3). These results suggest that BCG-induced MKP-1 expression is dependent on both p38 MAPK and ERK1/2.

### Transfection of MKP-1 siRNA into PBMo can reduce BCG-induced MKP-1 production

**Figure 4:**
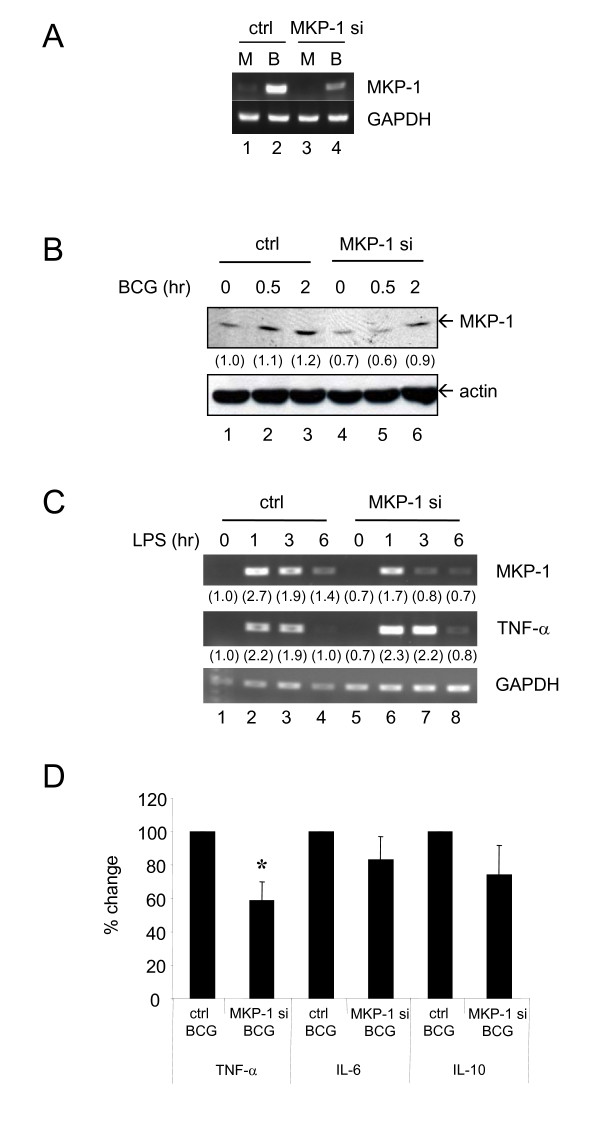
**Transfection of MKP-1 siRNA into primary human blood monocytes can increase LPS-induced TNF-α level while decrease BCG-induced TNF-α expression**. Cells were first transfected with control (ctrl) or MKP-1 siRNA (MKP-1 si, 200 nM) for 24 hours and then treated with different inducers as indicated. (A) After transfection, cells were treated with Mock (M) or BCG (B, MOI = 1 CFU/cell) for 3 hours. Levels of MKP-1 mRNA were measured by RT-PCR. (B) Monocytes were treated as in (A) for the indicated time points and MKP-1 proteins were measured by Western blotting. (C) Cells were treated with LPS (50 ng/ml) for the indicated time points. RNA was harvested, and levels of MKP-1 and TNF-α were measured by RT-PCR. For both (B) and (C), the intensities of the PCR/protein bands were determined by using Bio-Rad Quantity One imaging software. The intensities of bands were normalized to the corresponding control. The values in parenthesis are the relative normalized intensities compared to those of the control siRNA-transfected cells without other treatment. (D) BCG (MOI = 1 CFU/cell) was added for 3 hours. RNA was harvested and levels of TNF-α, IL-6 and IL-10 were measured by QPCR. Percentage change was defined as the percentage of the fold induction of (MKP-1 si + BCG) over the fold induction of (ctrl + BCG). Results of (ctrl + BCG) were set as 100%. For (A)-(C), independent experiments were done on monocytes from 3 different donors and one representative set of results is shown. For (D), experiments were performed on 4 different donors and the results are shown as mean ± SD. * = *p *< 0.05.

Throughout the above experiments, the primary goal was to examine the induction of MKP-1 by BCG in human monocytes. Thus, to further examine the role of MKP-1 in BCG-induced signaling, transfection of siRNA into PBMo was used to knockdown the activity of MKP-1. To demonstrate that the MKP-1 siRNA can indeed knockdown the target gene, PBMo were first transfected with control or MKP-1 siRNA and then treated with BCG for 3 hours. Levels of MKP-1 mRNA were measured by RT-PCR method. In Figure 4A, BCG stimulated MKP-1 expression (lanes 1 and 2). In MKP-1 siRNA transfected monocytes, induction of MKP-1 by BCG was significantly decreased (lanes 2 and 4). The results showed that the siRNA does abrogate the levels of MKP-1 mRNA.

To further determine whether MKP-1 siRNA affects BCG-induced MKP-1 at protein levels, PBMo were treated as above and MKP-1 proteins were measured by Western blotting. The results showed that BCG could induce MKP-1 proteins as usual for cells transfected with control siRNA (Figure 4B, lanes 1-3). However, the levels of BCG-induced MKP-1 protein expression were reduced in cells transfected with MKP-1 siRNA (Figure 4B, lanes 4-6). Together, the results suggest that MKP-1 siRNA not only reduced the MKP-1 mRNA in BCG treatment but also abrogated the BCG-induced MKP-1 protein.

### LPS-induced cytokine expression is increased by MKP-1 siRNA

As stated in the literature [9], MKP-1 KO mice showed increased TNF-α production in response to LPS. On the basis of the above MKP-1 siRNA results, LPS was then used as a control to demonstrate the effects of this MKP-1 siRNA system.

PBMo were first transfected with control or MKP-1 siRNA for 24 hours and then stimulated with LPS for the indicated time periods. Likewise, LPS induced MKP-1 within 1 hour treatment and the effects lasted for at least 6 hours (Figure 4C, lanes 1-4). The use of MKP-1 siRNA can reduce the levels of MKP-1 mRNA induced by LPS (Figure 4C, lanes 5-8). On the contrary, TNF-α levels were increased in MKP-1 siRNA transfected monocytes upon challenge with LPS (Figure 4C, lanes 5-8). The increases in cytokine expression induced by LPS in MKP-1 siRNA transfected cells suggest that the siRNA system is effective in knocking down the MKP-1 expression and MKP-1 acts as a negative regulator in LPS-induced TNF-α expression.

### BCG-induced cytokine expression is decreased by MKP-1 siRNA

To investigate the effect of MKP-1 siRNA on BCG-induced cytokine expression, the levels of TNF-α, IL-6 and IL-10 mRNA were measured by QPCR method. PBMo were transfected with either control or MKP-1 siRNA. Following exposure to BCG with control siRNA, there were significant inductions of TNF-α, IL-6 and IL-10 mRNA levels for 3 hours after treatment as previously reported ([5] and data not shown). Next, the effects of MKP-1 siRNA were examined on the cytokine expression induced by BCG. Surprisingly, there was a significant abrogation of BCG-induced TNF-α expression by MKP-1 siRNA (Figure 4D). With the knockdown of MKP-1, the level of BCG-induced TNF-α was only 60% compared to that of the control cells, while BCG-induced IL-6 and IL-10 were unchanged in MKP-1 siRNA transfected cells. The results revealed that MKP-1 plays a role in the induction of TNF-α expression upon BCG stimulation, which may be different from that of its conventional functions in which MKP-1 acts as a negative regulator in LPS-induced signaling pathways [7].

### BCG-induced MAPK phosphorylation is decreased by MKP-1 siRNA

**Figure 5:**
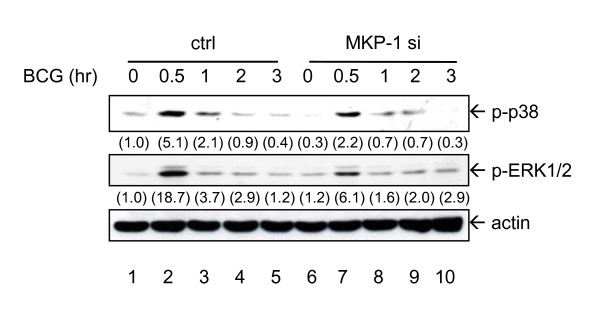
**BCG-induced MAPK phosphorylation is decreased by MKP-1 siRNA**. Transfection was done as in Figure 4, and BCG (MOI = 1 CFU/cell) was added for the indicated time points. Protein samples were harvested and levels of phospho-p38 MAPK (p-p38), phospho-ERK1/2 (p-ERK1/2) and actin were measured by Western blotting. Independent experiments were done on monocytes from 4 different donors and one representative set of results is shown. The intensities of the protein bands were determined by using Bio-Rad Quantity One imaging software. The intensities of phospho-proteins were normalized to the corresponding actin. The values in parenthesis are the relative normalized intensities compared to those of control siRNA-transfected cells without other treatment.

The unexpected observations in cytokine expression lead to the investigation on the effects of MKP-1 siRNA on BCG-induced MAPK activation. MKP-1 was found to have a preferential substrate binding to p38 MAPK and JNK than ERK1/2 [7]. The phosphorylation status of MAPKs was assessed in control or MKP-1 siRNA transfected PBMo. Western blotting results demonstrated that BCG-induced both p38 MAPK and ERK1/2 phosphorylation in 15 minutes (data not shown) and peaked at 30 minutes, and then returned to basal levels in cells treated with the control siRNA (Figure 5). Similar to the results of cytokine expression, phosphorylation of both p38 MAPK and ERK1/2 in response to BCG was decreased in monocytes transfected with MKP-1 siRNA instead of the expected increase in phosphorylation (Figure 5). The results suggest that MKP-1 knockdown would result in reduced MAPK phosphorylation by BCG, implying that the reduced level of TNF-α production in BCG stimulated monocytes is due to reduced phosphorylation of MAPKs by MKP-1 siRNA.

## Discussion

This report presented evidences that a novel function of MKP-1 is uncovered in cytokine regulation in response to mycobacterial infection. BCG induces MKP-1 as a rapid response (Figure 2). The induction mechanism of MKP-1 by BCG is dependent on both ERK1/2 and p38 MAPK (Figure 3). Using siRNA approach, the functions of MKP-1 can be examined in primary human monocytes. The results showed that the BCG-induced MAPKs activation as well as cytokine expression are downstream of MKP-1 (Figures 4D and 5). Thus, MKP-1 is a critical signaling molecule that is involved in BCG-induced cytokine expression.

Previous reports have shown that MKP-1 induced by LPS or peptidoglycan is dependent on p38 MAPK [14]. Accordingly, BCG-induced MKP-1 can be inhibited by both p38 MAPK and ERK1/2 inhibitors. Interestingly, it has been shown that degradation of MKP-1 is reduced after ERK1/2 phosphorylation [15]. It can be hypothesized that BCG-induced MKP-1 proteins can be stabilized by ERK1/2 and the detailed mechanisms involved require more exploration. Also, since the inhibition of MKP-1 expression by both inhibitors (for p38 MAPK and ERK1/2) was not complete, it is believed that other proteins may be involved in the BCG-induced MKP-1 expression.

**Figure 6:**
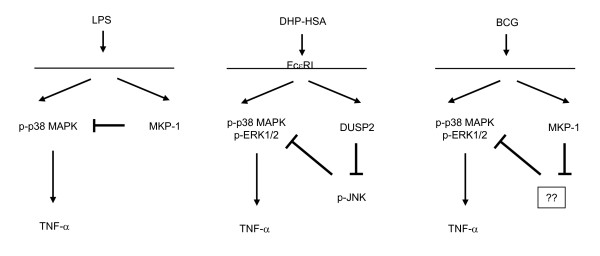
**MKP-1 plays a critical role in the regulation of cytokine expression upon mycobacterial infection**. LPS model was provided according to literature findings (Left). In this scenario, LPS activates MKP-1, which in turn dephosphorylates and deactivates phospho-p38 MAPK, resulting in less TNF-α induction. However, the situation in DHP-HSA activation of DUSP2 is more complicated (Middle), since the phosphatase activity causes subsequent inhibition of phospho-JNK which leads to the de-repression of phospho-p38 MAPK. Consequently, the combined effects of this cascade results in more TNF-α expression. The unexpected antimycobacterial role of MKP-1 (Right) may be explained by events similar to the DUSP2 effects. In this case (Right), there was an inhibition of unknown pathways or kinases downstream of MKP-1, and the unknown factor in turn inhibits MAPKs activation leading to more TNF-α induction. The details and kinase targets are yet to be identified.

On the basis of the literature results on LPS effects (Figure 6), the original expectation for this project is that MKP-1 acts as a negative regulator. LPS-stimulated MKP-1 KO peritoneal macrophages showed prolonged phosphorylation of p38 MAPK and JNK as well as increased production of TNF-α [9]. In doing so, LPS-induced MKP-1 could prevent prolonged TNF-α production as in sepsis which may lead to severe damage to the host. It was expected that BCG induces MKP-1 and its induction would correlate with the dephosphorylation of MAPKs including p38 MAPK. By blocking the MKP-1 using siRNA, it was expected to have increased p38 MAPK phosphorylation and prolonged TNF-α production in response to BCG. Nevertheless, our results shown here are diametrically opposite. One possibility for the unexpected results may be due to non-specific effects of transfection or siRNA. However, this was not the case since there was a prolonged and increased TNF-α expression after the MKP-1 siRNA-transfected monocytes were treated with LPS (Figure 4C).

There is now a new hypothesis to explain such paradoxical effects of MKP-1 in TNF-α regulation in which the phosphatase plays a role in positive regulation of TNF-α production in response to BCG as in the case of DUSP2 [13]. The structures of MKP-1 and DUSP2 are similar, with which they both contain a MAPK-interacting domain and a phosphatase catalytic site. By contrast, other DUSP may have extra domains, e.g., PEST [6]. Here, we postulate that the function of MKP-1 in BCG-induced signaling is similar to that of the DUSP2/PAC1.

Actually, the discovery of DUSP2 has initially created some paradoxical questions. As described, DUSP2 behaves differently from other MKP family members [13]. In DUSP2 KO macrophages treated with LPS, they produced less inflammatory mediators including less TNF, IL-6, nitric oxide, and IL-12-producing cells, when compared to that of the wild type counterparts [13]. Indeed, the results of these published studies on DUSP2 studies are quite similar to that of our reported results here.

It is plausible that these unexpected positive regulations of immune cell functions by DUSP2 were due to crosstalks between MAPKs [13]. It was shown that there are interactions between JNK and ERK1/2 pathways [16]. Here, we showed that the sustained activation of JNK blocks ERK activation (Figure 6). In the DUSP2 situation, stimulation of KO mast cells and macrophages shows increased phosphorylation of JNK, and inhibition of JNK by its own specific inhibitor restores phosphorylation of ERK1/2 [13].

In the BCG-MKP-1 situation, there is an early phosphorylation of p38 MAPK and ERK1/2. Therefore, it is possible that JNK may play a role in the crosstalk interaction of MAPK. However, our preliminary data suggest that the level of phosphorylated JNK was not increased in PBMo transfected with MKP-1 siRNA (data not shown). Thus, the details of the crosstalk between MAPKs need further investigation. Here, we present a model to summarize the results and to hypothesize the existence of an as yet unidentified intermediary factor or factors in the pathways downstream of MKP-1 effects in the BCG-induced signaling cascade. The unexpected antimycobacterial role of MKP-1 (Figure 6) may be explained by events similar to the DUSP2 effects. In this case, BCG induces MKP-1 expression while also activates MAPKs including p38 MAPK and ERK1/2. Downstream of MKP-1, there is an inhibition of unknown pathways or kinases. The unknown factor in turn inhibits MAPKs activation, which ultimately leads to more TNF-α induction (Figure 6).

## Conclusions

In summary, MKP-1 plays a critical role in the regulation of cytokine expression upon mycobacterial infection. Inhibition of unknown pathways or kinases downstream of MKP-1, which in turn inhibits MAPKs activation, may be used to explain the novel function of MKP-1 in enhancing MAPK activity and consequent TNF-α expression following BCG treatment (Figure 6). Taken together, the role of MAPK crosstalks need further exploration.

## Methods

### BCG

BCG vaccine Danish strain 1331 (Statens Serum Institut) was used in all experiments. The vaccine has passed the safety test, in which there are no macroscopic signs of tuberculosis in vaccinated animals, as stated in the Certificate of Analysis provided by Quality Assurance Department, Statens Serum Institut, Copenhagen, Denmark.

### Isolation of PBMo

PBMo were isolated from buffy coats of healthy blood donors (source: the Hong Kong Red Cross Blood Transfusion Service) by Ficoll-Paque (Pharmacia Biotech) density gradient centrifugation as before [17-19] with modification using MACS^® ^Technology according to the manufacturer's instructions (Miltenyi Biotec, Germany). Approval has been obtained from Hong Kong Red Cross Blood Transfusion Service for the use of donors' blood. Institutional Review Board of the University of Hong Kong has given exemption to this project since it did not involve the use of human or animal subjects. Briefly, the fresh leukocytes were centrifuged at 3000 r.p.m. for 15 minutes and were separated into plasma and cell layers. Plasma was removed and incubated at 56°C for 30 minutes. It was quickly chilled on ice and centrifuged at 3000 r.p.m. for 10 minutes. The supernatant was then filtered through syringe filter and used as the autologous plasma. The cell layer was diluted with PBS in the ratio of 1:1. The diluted cells were overlaid over the Ficoll very slowly and centrifuged at 2300 r.p.m. for 20 minutes. The lymphocyte/monocyte layer was removed and washed with RPMI medium 1640 until the supernatant was clear. The cell pellet was resuspended in PBS with CD14 MicroBeads (Miltenyi Biotec, Germany) and incubated at 4°C for 30 minutes. The cells were washed with PBS and passed through MACS^® ^LS Columns placed in a MACS^® ^Separator. The columns were washed with PBS for three times and were removed from the separator. PBMo were flushed out from the columns by applying the plunger. The cells were finally seeded onto the tissue culture plates in RPMI, supplemented with 5% autologous plasma. Cell purity of CD14^+ ^monocytes, measured by flow cytometry and antibody conjugated with PE against CD14 (Beckman, France), was >90%.

### Isolation of RNA

Total RNA extraction was done by using TRIzol reagent (Invitrogen, USA) according to the manufacturer's instructions. Cells were lysed in TRIzol reagent at room temperature for 5 minutes to allow for complete dissociation of nucleoprotein. Chloroform was then added. The tube was vigorously shaken for 15 seconds and incubated at room temperature for 3 minutes. The sample was centrifuged at 4°C for 15 minutes at 13,200 r.p.m. The colorless upper aqueous phase was transferred to a fresh tube. RNA was precipitated from aqueous phase by mixing with one volume of isopropyl alcohol at room temperature for 10 minutes. The sample was centrifuged again at 4°C for 10 minutes at 13,200 r.p.m. The supernatant was removed and the RNA pellet on the side and bottom of the tube was washed once with 75% ethanol. The sample was mixed by vortexing and centrifuged at 4°C for 5 minutes at 13,200 r.p.m. After removing the ethanol, the RNA pellet was dried in air for 10 minutes. Each RNA sample was dissolved in DEPC-water mixed with RNase inhibitor (Amersham Biosciences, USA). It was finally mixed with DNase buffer [25 mM Tris-HCl (pH 7.4), 5 mM MgCl_2_], 1 unit of DNase at 37°C for 15 minutes and then at 70°C for 10 minutes for deactivating the DNase. The samples were stored at -70°C until use.

### Reverse Transcription - Polymerase Chain Reaction (RT-PCR)

First-strand cDNA was reverse-transcribed in a 20 μl reaction. DNase-treated RNA and 0.5 μg oligo(dT) primer (Invitrogen, USA) were heated at 70°C for 10 minutes and chilled quickly on ice. The product was then mixed with first strand buffer (50 mM Tris-HCl [pH 8.3], 75 mM KCl, 3 mM MgCl_2_), 10 mM dithiothreitol (DTT), 0.5 mM deoxyribonucleoside triphosphates (dATP, dTTP, dCTP and dGTP) and 100 units of SuperScript™ II Reverse Transcriptase (Invitrogen, USA) at 42°C for 1 hour. The product was stored at -20°C until use for PCR and QPCR assays. Amplification of the reverse-transcribed cDNA was performed in a 25 μl reaction. PCR assays contained 5 pmol of each upstream and downstream primer, 1 unit of *Taq *DNA polymerase (Amersham Biosciences, USA), 0.2 mM each dNTP, and PCR reaction buffer [50 mM KCl, 1.5 mM MgCl_2_, and 10 mM Tris-HCl (pH 9.0)]. PCR were allowed to proceed for various cycles (94°C for 30 s, melting temperature (TM) for 30 s, and 72°C for 1 min). PCR primer sets used and assay conditions were as follows: (1) GAPDH, 25 cycles (TM = 60°C), upstream, 5'-ACCACAGTCCATGCCATCAC-3', downstream, 5'-TCCACCACCCTGTTGCTGTA-3'; (2) MKP-1, 35 cycles (TM = 52°C), upstream, 5'-GCTGTGCAGCAAACAGTCGA-3'; downstream, 5'-CGATTAGTCCTCATAAGGTA-3' and (3) TNF-α, 30 cycles (TM = 56°C), upstream, 5'-GGCTCCAGGCGGTGCTTGTTC-3', downstream, 5'-AGACGGCGATGCGGCTGATG-3'. PCR products were analyzed on a 1% agarose gel with ethidium bromide and visualized under ultraviolet light. In order to check the size of the PCR products, 1 kb Plus DNA Ladder™ (Invitrogen, USA) was run along with the PCR products.

### Quantitative polymerase chain reaction (QPCR)

To perform QPCR, the levels of MKP-1, and TNF-α mRNA as well as the reference gene GAPDH (as internal control) were assayed by the gene-specific Assays-on-Demand reagent kits (Applied Biosystems, USA). All samples were run in duplicates or triplicates and with no template controls on an ABI Prism 7700 Sequence Detector. The analysis method of QPCR was the comparative cycle number to threshold (C_T_) method as described in user bulletin no. 2 of the ABI Prism 7700 Sequence Detection System. The number of C_T _of the targeted genes was normalized to that of GAPDH in each sample (ΔC_T_). The C_T _value of the treated cells was compared with that of the untreated or mock-treated cells (ΔΔCT). The relative gene expression of the targeted genes (fold induction) was calculated as 2^-ΔΔCT^.

### Protein extraction

Total cellular proteins were extracted by lysing cells in lysis buffer containing 1% Triton X-100, 0.5% NP-40, 150 mM NaCl, 10 mM Tris-HCl (pH 7.4), 1 mM EDTA, 1 mM EGTA (pH 8.0), 1% SDS, 0.2 mg/ml PMSF, 1 μg/ml aprotinin, 1 mM sodium orthovanadate, 2 μg/ml pepstatin, 2 μg/ml leupeptin, and 50 mM sodium fluoride for 5 minutes. The homogenate was then boiled for 10 minutes and stored at -70°C until use. The concentrations of total protein in cell extracts were determined by BCA™ Protein Assay Kit (Pierce, IL, USA).

### Western blot analysis

Western blot was done as described [20]. Equal amounts of protein were separated by 10% SDS-PAGE, electroblotted onto nitrocellulose membranes (Schleicher & Schuell), and followed by probing with specific antibodies for Actin, MKP-1 (Santa Cruz Biotech., USA), phospho-p38 MAPK, phospho-ERK1/2 (Cell Signaling, USA). After three washes, the membranes were incubated with the corresponding secondary antibodies. The bands were detected using the Enhanced Chemiluminescence System (Amersham Pharmacia Biotech) as per the manufacturer's instructions.

### Transfection of siRNA

Transfection of siRNA into human monocytes was done as described [21]. MKP-1 siRNA included (i) MKP1-HSS102982, AAACGCUUCGUAUCCUCCUUUGAGG; (ii) MKP1-HSS102983, UUAUGCCCAAGGCAUCCAGCAUGUC; and (iii) MKP1-HSS102984, UGAUGGAGUCUAUGAAGUCAAUGGC. MKP-1 knockdown in PBMo was conducted by using MKP1-HSS102983 only or a pool of the above three different MKP-1 Stealth™ Select RNAi (ratio = 1:1:1, 200 nM, Invitrogen, USA). Stealth™ RNAi Negative Control Duplex (200 nM) was used as a control for sequence independent effects for the siRNA transfection. Transfection of monocytes was done by using jetPEI™ DNA transfection reagent (Polyplus Transfection, USA) according to the manufacturer's instructions. After transfecting the cells for 24 h, the transfectants were treated with different inducers as described above.

### Statistical Method

Statistical analysis was performed by Student's *t *test. Differences were considered statistically significant when *p *values were less than 0.05.

## List of abbreviations

BCG: Bacillus Calmette Guerin; ERK1/2: extracellular signal-regulated kinase 1 and 2; GAPDH: gyceraldehyde-3-phosphate dehydrogenase; IL: Interleukin; KO: Knock-out; LPS: Lipopolysaccharide; MAPK: mitogen-activated protein kinase; MKP: MAPK phosphatase; MOI: multiplicity of infection; MTB: *Mycobacterium tuberculosis*; PBMo: human peripheral blood monocytes; PE: Phycoerythrin; SAPK/JNK: stress-activated protein kinase/c-Jun N-terminal kinase; si: short interference; TB: Tuberculosis; TBS-T: Tris-buffered saline-Tween; TNF-α: tumor necrosis factor-α

## Authors' contributions

BKWC and NCML were involved in performing the experiments while all authors participated in the design of experiments, analyzed results, and wrote and approved the manuscript.
